# Blink Rate Patterns Provide a Reliable Measure of Individual Engagement with Scene Content

**DOI:** 10.1038/s41598-020-64999-x

**Published:** 2020-05-19

**Authors:** Carolyn Ranti, Warren Jones, Ami Klin, Sarah Shultz

**Affiliations:** 1 Marcus Autism Center, Children’s Healthcare of Atlanta, Atlanta, Georgia 30329 USA; 2 Division of Autism & Related Disabilities, Department of Pediatrics, Emory University School of Medicine, Atlanta, Georgia 30022 USA; 3 Center for Translational Social Neuroscience, Emory University, Atlanta, Georgia 30022 USA

**Keywords:** Attention, Human behaviour

## Abstract

Eye-blinking has emerged as a promising means of measuring viewer engagement with visual content. This method capitalizes on the fact that although we remain largely unaware of our eye-blinking in everyday situations, eye-blinks are inhibited at precise moments in time so as to minimize the loss of visual information that occurs during a blink. Probabilistically, the more important the visual information is to the viewer, the more likely he or she will be to inhibit blinking. In the present study, viewer engagement was experimentally manipulated in order to: (1) replicate past studies suggesting that a group of viewers will blink *less* often when watching content that they perceive as *more* important or relevant; (2) test the reliability of the measure by investigating constraints on the timescale over which blink rate patterns can be used to accurately quantify viewer engagement; and (3) examine whether blink rate patterns can be used to quantify what an *individual* – as opposed to a *group* of viewers—perceives as engaging. Results demonstrate that blink rate patterns can be used to measure changes in individual and group engagement that unfold over relatively short (1 second) and long (60 second) timescales. However, for individuals with lower blink rates, blink rate patterns may provide less optimal measures when engagement shifts rapidly (at intervals of 1 second or less). Findings support the use of eye-blink measures in future studies investigating a person’s subjective perception of how engaging a stimulus is.

## Introduction

In everyday complex environments, successful adaptive action depends upon selectively engaging with things that have the greatest behavioral relevance. Information that is not perceived as relevant—even if looked at—may go unprocessed^1,2^. For this reason, engagement—defined herein as a kind of focused attention that involves investment or engrossment in an activity or with a person or thing^3–5^—is a critical gating mechanism for successful learning^1,2^. The current study investigates a novel measure of a specific facet of engagement: perceived stimulus salience, defined herein as a person’s subjective perception of how important or engaging a stimulus is. Perceived stimulus salience guides attention towards what is perceived to be most important at any given moment and, consequently, acts as a gatekeeper in selecting experiences that will directly affect learning, memory, ongoing brain activation, and subsequent brain specialization^6–9^.

The perceived salience of a stimulus can be driven by a host of interacting factors, including the physical properties of the stimulus^10^ and the internal needs and states (i.e., the goals, motivations, and interests) of the viewer^11–14^. Perceived stimulus salience is therefore a *dynamic* property that changes over space and time (varying as a function of changes in stimulus properties or changes in a viewer’s internal needs), and an inherently *subjective* aspect of viewer experience. Consequently, viewers’ assessment of the salience of a stimulus—i.e., real-time appraisals of what is perceived *by the viewers themselves* as being important or salient to process—has traditionally been very difficult to quantify objectively and with high temporal resolution, limiting scientific inquiry into this critical phenomenon.

Recently, however, eye-blink inhibition has emerged as a promising means of measuring the perceived salience of scene content. This method capitalizes on the fundamental tradeoff between the physiological benefits of blinking—keeping the eye lubricated and cleansed^15^—and the potentially negative consequences of the loss of visual information that occurs during a blink^16–18^. Given this tradeoff, it would be highly adaptive to dynamically adjust the exact timing of when we do or do not blink. Indeed, although we remain largely unaware of our own eye-blinking in everyday situations, the precise timing of eye-blinking is dynamically, unconsciously adjusted^18–25^: probabilistically, viewers are least likely to blink when looking at something perceived to be most important. Consequently, viewers’ subjective perception of stimulus salience can be quantified by measuring their rate of eye-blinking while viewing visual information.

This method was first described by our laboratory in a study examining whether the timing of eye-blinking during natural viewing varies as a function of viewer engagement^26^. This hypothesis was tested by collecting eye-tracking and eye-blink data from two groups of viewers with very different interests: typically-developing toddlers and toddlers with Autism Spectrum Disorder (ASD). Unlike typically-developing toddlers, toddlers with ASD show reduced attention to a range of social cues and increased attention to physical cues^26–33^. To capitalize on the different internal goals and interests of these two groups of children, we presented them with a video of an unscripted, naturalistic interaction that included both physical movements (the opening and closing of a door on a toy wagon) and affectively charged social interactions (an argument between two onscreen characters). As expected, typical 2-year-olds *inhibited* their blinking when watching emotionally charged scenes and when looking at the faces of onscreen characters, whereas 2-year-olds with ASD inhibited their blinking when looking at physical objects and at physical objects in motion. These results suggest that by measuring the timing of blink inhibition relative to unfolding scene content, one can determine, on a moment-by-moment basis, viewers’ unconscious, subjective appraisals of the importance of what they are watching.

Several studies by independent research groups have also investigated the relationship between eye-blinking and cognitive processes, demonstrating that: (1) eye-blinking *decreases* during the presentation of task-relevant stimuli in both visual and auditory domains^34,35^; (2) eye-blinking *predicts* how strongly movie scenes will be remembered (with moments of *decreased blinking*—i.e., increased engagement—coinciding with content that is more strongly remembered, evidence of a remarkably direct link to learning)^36^; (3) eye-blinking *increases* during moments when the probability of missing important visual information is low (i.e., during *less* engaging moments)^19^; and (4) eye-blinking is similarly decreased at times of increased vigilance and alertness in multiple non-human species^37,38^. While these studies used varying terminology to describe the cognitive processes that influence eye-blinking, the constructs invoked share key features of engagement (engrossment or investment in features of the environment^3,4,39^), providing additional support for the notion that eye-blinking provides a marker of engagement in both human and non-human species.

Given the potential of measures of eye-blinking for indexing perceived stimulus salience, the goal of the current paper is to further validate the utility of eye-blinking as a measure of engagement and to test the reliability of the measure under varying conditions. Unlike our previous study in which perceived salience was defined by the viewers’ own internal goals and interests (i.e., the different interests of typical toddlers and toddlers with ASD), the current study experimentally manipulates perceived stimulus salience by randomly assigning participants to engage with one of two tasks while watching the same stimuli, providing a test of the hypothesis that viewers will blink *less* often when watching content perceived as being *more* important or relevant to process.

We also investigate whether there are constraints on the *timescale* over which blink rate patterns can be used to reliably quantify viewer engagement. To take an extreme example, we would expect that measures of eye-blinking would necessarily fail to detect changes in engagement that unfold more quickly than the duration of an eye-blink (typically 150–400 ms^25,40^). At the other extreme, blink rates averaged over the course of days or weeks may fail to identify relatively brief fluctuations in engagement. To determine whether blink rate patterns can resolve differences in perceived stimulus salience across varying timescales, the current study explicitly manipulates viewer engagement with scene content over timescales ranging from 1 to 60 seconds in duration. This specific range of relatively short timescales was selected because of how quickly the dynamics of visual scene processing can unfold^41,42^.

Finally, we test whether blink rate patterns can be used to quantify what an *individual* – as opposed to a *group* of viewers—perceives as engaging. While previous research has used blink rate patterns to quantify what is perceived as engaging by groups^26^, quantifying what an individual perceives as engaging presents unique challenges. For instance, individual viewers spend far more time *not* blinking than blinking, with healthy adults averaging approximately 4 to 30 blinks per minute^43–46^. Given the relative sparseness of individual relative to group eye-blink data, it may be more challenging to determine whether a lack of eye-blinking on the part of an individual reflects increased engagement or whether it simply reflects the absence of a physiological need to blink. This challenge may be especially pronounced among individuals with lower blink rates^44^. The present study examines whether individual (as opposed to group) blink rates can be used to recover information about an individual’s assessment of perceived stimulus salience, and, if so, whether the utility of individual measures of engagement varies as a function of individual blink rates.

**Figure 1:**
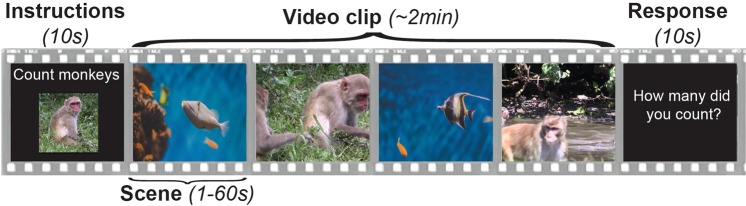
Task schematic. At the beginning of each composite video, participants saw an instruction screen for 10 s, indicating the animal to be counted in the upcoming video. Then, they saw a video that alternated between scenes of animals under water and scenes of animals on land. Across the 13 timing conditions, the alternating scene time (duration of land scene shown before a water scene was shown or vice versa) was between 1 s and 60 s in length. All scenes in a composite video were the same length. Finally, participants saw a 10 s response screen, prompting them to report the number of target animals counted. The 13 composite videos were presented in random order.

In order to experimentally manipulate perceived stimulus salience, we randomly assigned typical adults (n = 21) to engage in one of two tasks while watching the same stimuli. All participants watched videos that alternated between scenes of animals on land and scenes of animals under water at a constant rate of 1, 5, 10, 15, 20, 25, 30, 35, 40, 45, 50, 55, or 60 s, for a total of 13 composite videos (1 for each timing condition) (see Fig. 1). Half of the participants were instructed to count the number of land animals in each scene, and the others were instructed to count the number of water animals in each scene (participant groups are referred to henceforth as ‘land counters’ and ‘water counters’, respectively). As a result of this task assignment, the two categories of scene content (animals on land and animals under water) were experimentally manipulated to be differentially engaging: by task design, land scenes will be more engaging to ‘land counters’ whereas water scenes will be more engaging to ‘water counters’.

Eye-tracking data were collected while participants watched the videos, and blinks were identified by an automated algorithm measuring occlusion of the pupil by rate of change in pupil diameter and by vertical displacement of the measured pupil center, as in Shultz *et al*.^26^. Mean blinks per minute were calculated for each participant during: (1) the entire viewing session; (2) the land animal scenes; and (3) the water animal scenes.

## Results

### Modulation of group blink rate by experimental task

**Figure 2:**
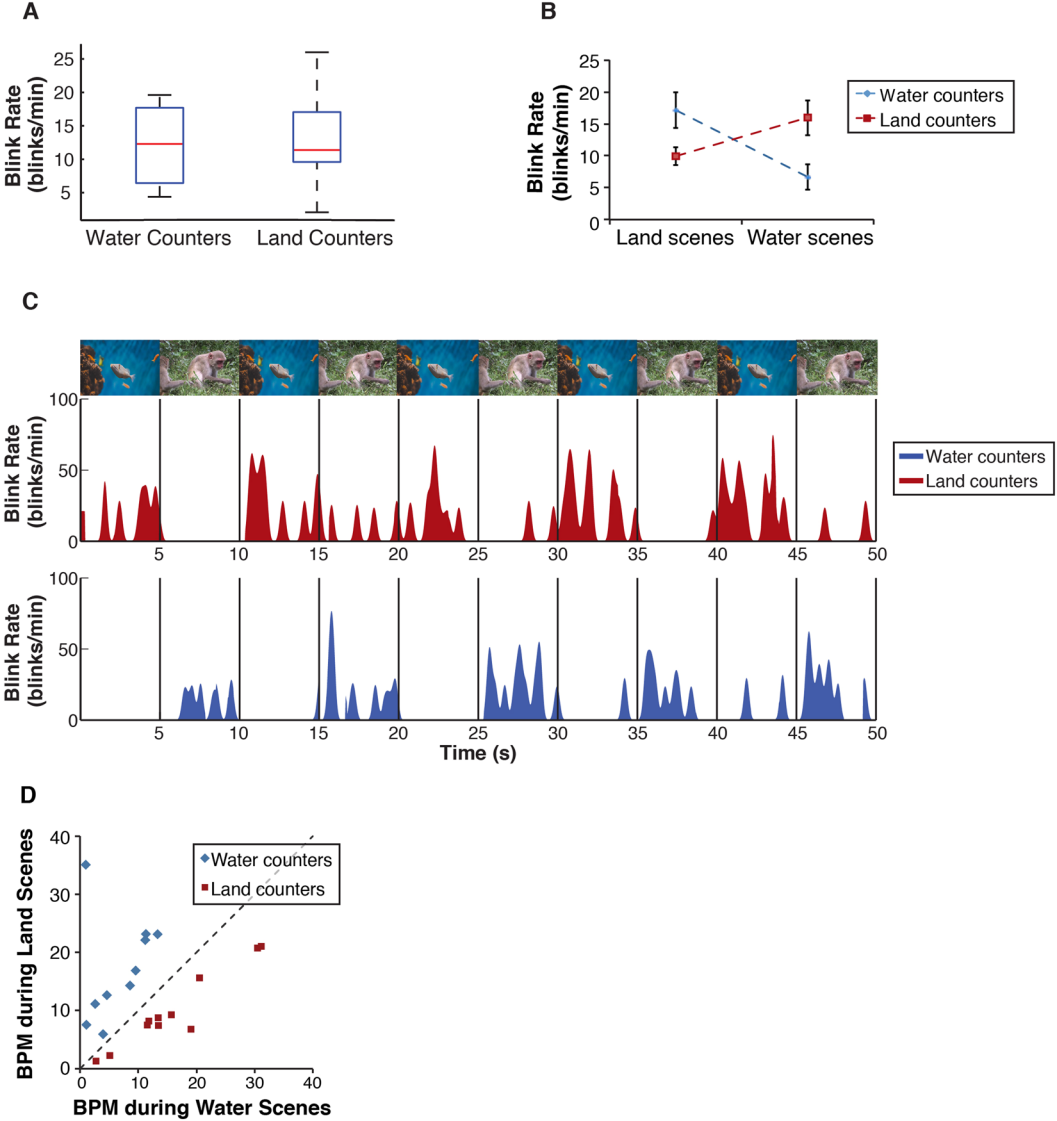
Blink rate by experimental group and condition. (**A**) Overall blink rate: Participants randomly assigned to one of two experimental tasks (land counters or water counters) did not differ in their average blink rate over the entire viewing session. (**B**) Blink rate by task condition: There was a significant interaction between scene type and experimental group on blink rate: both land and water counters blinked less often during scenes that contained content relevant to their assigned task. (**C**) Instantaneous blink rate (exemplars): Smoothed instantaneous blink rate of water counters (blue) and land counters (red) while viewing a composite video alternating between water and land scenes (example shown alternates every 5 seconds). Group blink rates decreased during scenes that contained content relevant to the participants’ assigned task (i.e., blink rates decreased during land scenes for land counters and decreased during water scenes for water counters); likewise, group blink rates increased during scenes that did not contain task-relevant content (i.e., water scenes for land counters and vice versa). (**D**) Every participant had lower mean blinks per minute (BPM) during scenes that contained content relevant to the participant’s assigned task (i.e., land scenes for land counters and water scenes for water counters) and higher BPM during scenes that did not contain task-relevant content (i.e., water scenes for land counters and vice versa). Dashed diagonal line represents equal BPM during land and water scenes.

As expected, there was no difference in overall blink rate between land counters and water counters during the entire viewing session (two-sample t-test, t(19) = 0.28, *p* = 0.78; Shapiro-Wilk tests indicated that blink rate was normally distributed for both water and land counters (W(10) = 0.91, *p* = 0.27 and W(11) = 0.91, *p* = 0.23, respectively)) (Fig. 2A). To examine whether blink rate was modulated as a function of task, we compared blink rates for each experimental group (land counters or water counters) during land and water scenes. A mixed ANOVA with scene type (land vs water) as a within-subjects factor and experimental group (land counters vs water counters) as a between-subjects factor revealed a significant interaction between scene type and experimental group on blink rate (F(1,19)=33.903, *p* = 0.000013; Shapiro-Wilk tests indicated that blink rate was normally distributed for each combination of the within- and between-subjects factors, all *p*’s > 0.05.) (Fig. 2B). Paired t-tests revealed that viewers assigned to the ‘land counter’ group spontaneously *decreased* their blink rate during land scenes and *increased* their blink rate during water scenes (t(10) = −5.97, *p* = 0.00014). Similarly, the ‘water counter’ group spontaneously *decreased* their blink rate during water scenes and *increased* their blink rate during land scenes (t(9) = 3.79, *p* = 0.004). This pattern was consistent even on an individual basis: every participant had a lower blink rate (measured as mean blinks per minute) during scenes that contained content relevant to their assigned task (Fig. 2D).

### Measuring group engagement over varying timescales

**Figure 3:**
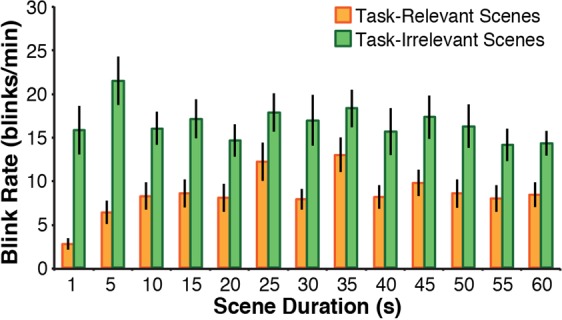
Measuring group engagement over varying timescales. Group blink rates for videos that alternated between land and water scenes at varying timescales (1–60 s). Across all timescales examined, participants blinked less during scenes that contained task-relevant content (‘task-relevant scenes’) and more during scenes that did not contain task-relevant content (‘task-irrelevant scenes’).

To investigate whether there are constraints on the timescale at which blink rates can be used to quantify viewer engagement, participants were presented with videos that alternated between land scenes and water scenes over varying timescales (*i.e*., presented with varying scene alternation rates of 1 scene per 1, 5, 10, 15, 20, 25, 30, 35, 40, 45, 50, 55, or 60 s). Group blink rates were calculated for each timing condition separately. Across all timescales examined, paired t-tests revealed that participants blinked *less* during scenes that contained content relevant to their assigned task (all *p*’s < 0.05, corrected for multiple comparisons using the Bonferroni-Holm method^47^) (Fig. 3).

### Classification of group membership using individual blink rate patterns

To investigate whether individual (as opposed to group) blink rate patterns can be used to recover information about perceived salience, we tested whether individuals could be correctly classified as having been assigned to the land or water counter condition on the basis of their blink rates during video watching.

**Figure 4:**
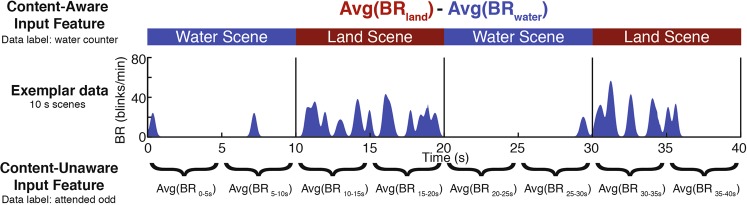
Example classification inputs for a single example participant assigned to the ‘water counter/attended odd’ condition. The input used for content-aware classification (top) was the participant’s average blink rate during all land scenes minus their average blink rate during all water scenes. This was calculated using data from all composite videos, and the participant was labeled as a ‘water counter’. For content-unaware classification (bottom), the input was a vector of the participant’s average blink rate during consecutive intervals (5 s intervals shown here), concatenated across all composite videos. The participant was labelled as ‘attended odd’.

Two separate classification analyses were performed, each using different input features (i.e., a different metric upon which classification was made). The first classification was made on the basis of the difference between a viewer’s mean blinks per minute during land and water scenes. The second classification was made on the basis of a viewer’s blinks per minute averaged over consecutive intervals throughout the composite videos (see Fig. 4). The critical distinction between these two input features is the extent to which they rely on knowledge of the task structure. The first requires knowledge of when task relevant and task irrelevant information was presented, while the second does not, making it more well-suited for paradigms in which moments likely to be perceived as engaging are not known a priori. These two classification analyses will be referred to henceforth as ‘content-aware’ and ‘content-unaware’ classification, respectively.

#### Content-aware classification

As described above, the input feature used for this classification was a participant’s average blink rate during all land scenes minus their average blink rate during all water scenes (see Fig. 4**)**. This input feature was calculated using data from all 13 videos. Each participant was labeled by experimental group (‘land counter’ or ‘water counter’). Sensitivity (the percentage of land counters correctly assigned to the land condition) and specificity (the percentage of water counters correctly identified as *not* being assigned to the land condition) were calculated across all possible discrimination threshold values. Receiver Operating Characteristic (ROC) curve analysis was used to plot the true positive rate (sensitivity) against the false positive rate (1 – specificity). The area under the empirical curve (AUC) is used to combine both dimensions into a single metric^48^.

Consistent with the finding that every participant blinked *less* during scenes that contained content relevant to their assigned task (see Fig. 2D), the AUC was 1, an indication of perfect classifier success. Sensitivity and specificity at the optimal threshold were 100%. Ninety-five percent confidence intervals for sensitivity and specificity (indicating the range of classification performance values one might expect to obtain if the same experiment was repeated on numerous samples^49^) were 67.9% to 100% and 70.0% to 100%, respectively.

#### Content-unaware classification

Unlike the content-aware classification analysis, in which classification was made on the basis of blink rates during land and water scenes, content-unaware classification was made on the basis of features that require *no* a priori knowledge of the task structure. Specifically, content-unaware classification was made on the basis of average blink rates during consecutive intervals of a fixed length, concatenated across all composite videos (see Fig. 4 for an example of content-unaware input features defined using 5 second intervals).

Given that there is no gold standard for selecting the optimal interval size over which to average blink rates, a range of plausible interval sizes were first identified by visual inspection. These intervals appeared to meaningfully capture changes in blink rate, while averaging over fluctuations that would be better characterized as noise. Classification was then performed over the entire range of plausible interval sizes, for a total of 10 analyses (10 interval sizes, evenly spaced between 1 and 10 seconds), to ensure that classification results were not influenced by choice of interval size.

For each composite video, blink rates were calculated during each interval size (1 to 10 seconds), and then concatenated across all 13 videos. These vectors were used to train a Support Vector Machine (SVM), a classification approach that uses supervised learning^50^. Given a set of training examples, each labeled as belonging to one of two categories, the SVM algorithm outputs the hyperplane that has the largest distance to the nearest training-data point of any category. This model is then used to assign new examples to one category or the other.

Data from each participant was labeled according to when the participant was actively counting animals in each of the videos (‘attended odd’ or ‘attended even’). For example, a water counter who saw the set of videos in which water scenes were presented *first* and then in every *odd*-numbered scene thereafter was labeled ‘attended odd’ (see Fig. 4 for example). By contrast, a water counter who saw the other set of stimuli, in which a water scene was presented *second* and then in every *even*-numbered scene thereafter, was labeled ‘attended even’.

Training and classification were performed using MATLAB’s ‘svmtrain’ and ‘svmclassify’ functions (Matlab R2015a), with a linear kernel. The classifier was trained and tested using a leave-one-out cross-validation procedure, whereby the classifier was trained on all the data minus one ‘left-out’ participant and then tested on the ‘left-out’ participant^51^. The regularization parameter (which characterizes the degree of importance that is given to miss-classifications) was selected for each training set by iterating over a range of potential values (10^−2^ to 10^10^)^52,53^. For each parameter value that was tested, a classifier was trained on 75% of the training set and then tested on the remaining 25% of the training set. The regularization parameter that produced the most accurate classifier was then used to train a classifier using the entire training set. This final classifier assigned a group label (‘attended odd’ or ‘attended even’) to the single excluded participant. The entire procedure was repeated for each participant.

Permutation testing was used to test the null hypothesis that classifier performance did not differ from chance^54^. In each of 1000 iterations, experimental group labels (‘attended odd’ and ‘attended even’) were randomly assigned without replacement to the 21 participants, and then the classifier was trained and tested in the manner described above. The 95^th^ percentile across all 1000 iterations served as a cutoff for statistically significant classifier success rate (*p* < 0.05).

The content-unaware classification approach was successful in classifying individuals as having been assigned to the land or water counter condition on the basis of their blink rates. At least 19 out of 21 participants were correctly classified for every interval size used (Table 1). All classification results differed from chance (all *p’s* < 0.01, assessed via permutation testing).

**Table 1:** Content-unaware classification results for each interval size.

Interval Size (s)	Percent Correct
1	90.48
2	95.24
3	95.24
4	95.24
5	95.24
6	90.48
7	90.48
8	90.48
9	95.24
10	100.00

### Influence of inter-individual variability in blink rates on individual measures of engagement

Consistent with previous reports^44,55^, high inter-individual variability in blink rates was observed in the present study (range, 2–26 blinks per minute (bpm); mean = 12.6 bpm; standard deviation = 6.7 bpm). To test whether the utility of individual measures of engagement varies as a function of individual blink rates, we examined the relationship between an individual’s blink rate and the strength of their assignment to their actual experimental group by the content-aware classifier.

For trials of every scene duration, kernel density estimation^56^ was used to calculate two probability density functions (PDFs): one for each experimental group’s content-aware classification metric (a viewer’s mean blinks per minute during all land scenes minus their mean blinks per minute during all water scenes). The PDFs define the probability of a given classification metric occurring in each experimental group. Then, classification strength was quantified for each participant by computing a likelihood ratio, defined as:

$$LR=\frac{P(participant\,classification\,metric\,occurring\,in\,correct\,group)}{P(participant\,classification\,metric\,occurring\,in\,incorrect\,group)}$$

This ratio compares the likelihood of the participant’s classification metric occurring in the distribution of the correct experimental group to the likelihood of the classification metric occurring in the distribution of the incorrect experimental group. Thus, an LR greater than 1 indicates that the metric is more likely under the correct experimental group. The higher the likelihood ratio, the better that individual fits their correct experimental group.

**Figure 5:**
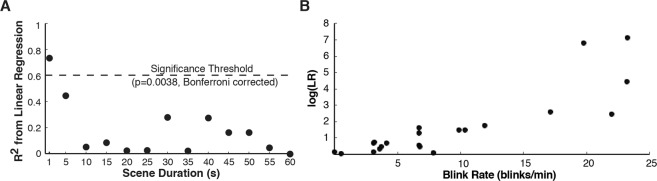
Relationship between individual blink rate and classification strength for scenes of varying length. (**A**) A significant relationship between individual blink rate and log likelihood ratio (log(LR)) was observed for 1 s scenes only. (**B**) The relationship between log(LR) and blink rates during 1 s scenes, indicating that classification strength (indexed by log(LR)) was lower for participants with lower blink rates.

Regression analyses were run to examine the relationship between an individual’s blink rate during a video and their log transformed LR. Linear, quadratic, cubic, and exponential regressions were run on the data for each scene duration, and p-values were Bonferroni corrected for multiple comparisons (13 total)^47^. Only the linear regression for the shortest scene duration (1 s) was significant (r^2^ = 0.735, *p* = 6.8319E-7) (see Fig. 5A and Supplementary Materials Fig. 1). Examination of the relationship between individual blink rates and log transformed LR during the composite video that alternated between land and water scenes every 1 s, revealed that strength of classification (indexed by LR) was *lower* for individuals with *lower* blink rates (see Fig. 5B).

Deming regression analyses (which, unlike linear regression, accounts for measurement errors in both individual blink rate and log transformed LR variables) confirmed the robustness of linear regression results (see Supplementary Materials Table 1 for details).

## Discussion

The present findings provide strong evidence that blink rate is modulated by engagement. Unlike our previous study, which examined natural variations in perceived salience during free-viewing of social scenes^26^, the present study used an experimental task to manipulate perceived salience by randomly assigning participants to engage with one of two tasks. Despite viewing the exact same visual stimuli, our results revealed that participants assigned to the ‘land counter’ and ‘water counter’ groups showed distinct blink rate patterns, with each group blinking *less* while viewing content perceived to be *more* salient and *more* relevant to their assigned task.

Our findings also demonstrate that blink rate patterns can be used to accurately quantify changes in perceived stimulus salience that unfold over a range of timescales. When viewers’ level of engagement with visual content was manipulated to change at varying rates (with scenes containing task-relevant content appearing every 1 to 60 seconds), each group still blinked *less* while viewing content perceived to be *more* salient or relevant to their assigned task. This suggests that blink rate patterns can be used to accurately measure changes in viewer engagement that unfold over relatively short (e.g., 1 s) and long (e.g. 60 s) timescales. Future studies are needed to assess the utility of eye-blink measures of engagement across even shorter and longer timescales.

While past work has demonstrated that *group* blink rates are significantly modulated by perceived stimulus salience, the present study provides the first demonstration that *a single viewer’s* blink rate patterns can be used to quantify how engaged they are with what they are looking at. Across the entire experiment, every individual’s blink rate was modulated by perceived salience: all participants blinked less frequently during scenes that contained content relevant to their assigned task. Furthermore, two classification analyses (content-aware and content-unaware classification) showed that a single viewer’s blink rate pattern contains sufficient information to correctly classify the viewer as having been assigned to the land counter or water counter group.

Unlike content-aware classification, which required knowledge of the task structure (i.e., when task relevant and task irrelevant scenes were presented), content-unaware classification was performed without relying on *a priori* knowledge of when participants were likely to be engaged. While both classification approaches were highly successful (supporting the utility of eye-blinking as an individual measure of engagement), the success of content-unaware classification is a promising indicator that group membership can be recovered on the basis of blink rates, even during more ecologically-valid, free-viewing paradigms, where moments perceived as engaging are not known *a priori*. For example, this method could be used to evaluate whether an individual’s blink rate pattern was more consistent with that of typically-developing viewers or viewers with a clinical condition, such as Autism Spectrum Disorder (ASD), during open-ended free viewing of naturalistic social scenes.

Finally, we examined whether the utility of individual measures of engagement varies as a function of individual differences in blink rate. Individual overall blink rates showed no relationship to classification strength (i.e., how closely their blink rate pattern matched that of their experimental group) for videos that alternated between land and water scenes every 5 to 60 seconds. This suggests that changes in an individual’s rate of eye-blinking over the course of a viewing session can be used to measure differences in viewer engagement that unfold over timescales of 5 to 60 seconds, even amongst viewers with very low mean blink rates (i.e., 2 blinks per minute). However, we did observe a significant relationship between blink rates and classification strength during videos that alternated most rapidly (i.e., every 1 second) between land and water scenes. This indicates that when viewer engagement changes rapidly (i.e., at intervals of 1 second or less), it may be more difficult to classify individuals who do not blink as often.

While our results indicate that change in a viewer’s blink rate over the course of a viewing session can reveal information about engagement, future research is needed to determine whether a viewer’s instantaneous blink rate can be used to index moment-by-moment variations in engagement. Given that individual viewers spend more time not blinking than blinking, it may be challenging to determine whether any one period of not blinking is indicative of engagement. However, a recent computational model that captures individual blinking behavior as a trade-off between an internal, physiological need to blink and the external task requirements of not blinking when task-related information needs to be acquired, suggests that such a measure may be possible, as long as some properties of the environmental statistics are known^18^. In addition, future studies should examine whether blink rate patterns can provide information about the extent to which viewers are engaged with what they are looking at. Given that rates of eye-blinking cannot decrease below zero, measures of eye-blinking may not be well-suited for capturing further increases in engagement once a certain level of engagement has been reached.

## Conclusions

The present findings demonstrate that blink rate patterns provide a reliable measure of viewer engagement. In particular, the finding that *individual* blink rate patterns contain enough information to correctly classify group membership has important implications for many different fields in which a viewer’s subjective perception of stimulus salience is an important aspect of investigation. For instance, in education and intervention programs, engagement has been identified as a fundamental mechanism of human learning and is a critical mediator of successful learning in individuals with developmental disabilities^1,57,58^. While there are currently no quantitative and objective measures of engagement for evaluating such programs^59^, the performance-based individualized measures of engagement described herein could be used to develop objective tools for quantifying this critical active ingredient for successful learning. In addition, in clinical research, measures of how engaged a viewer is with specific types of visual content could provide a biomarker of disease state, disease progression, and/or treatment response in conditions that affect engagement with circumscribed content or attentional biases, such as ASD^60^, schizophrenia^61^, bipolar disorder^62^, or depression^63^. Ongoing research in our laboratory is investigating whether blink rate patterns can be used to classify individuals by diagnostic group and to identify what types of content individual viewers with developmental disabilities, such as ASD, perceive as being highly engaging.

## Methods

### Participants

Twenty-one adults (mean age: 27.9 years, standard deviation: 6.8 years, range: 22–46 years; 3 male) with normal or corrected-to-normal vision participated in the study. One additional participant was excluded because the participant fell asleep during the session. All participants gave written informed consent, and the Emory Institutional Review Board approved the protocol. All methods were carried out in accordance with the relevant guidelines and regulations.

### Stimuli

Stimuli consisted of video footage that alternated between scenes of animals on land and scenes of animals under water (see Fig. 1). The animal scenes were drawn from various sources, including live feeds from the National Aquarium in Baltimore, a live feed from Zoo Atlanta, YouTube videos, and original footage recorded in Cayo Santiago, Puerto Rico.

Composite videos ranged from 1.5 to 2.67 minutes in length. Each composite video alternated between land scenes and underwater scenes at a constant rate of 1, 5, 10, 15, 20, 25, 30, 35, 40, 45, 50, 55, or 60 s, for a total of 13 video timing conditions. Each composite video contained scenes that were pulled from two source videos – one that depicted animals on land, and one that depicted animals under water. Participants never saw the same scene more than once, and all scenes were continuous shots with no abrupt changes in camera angles. Each participant saw all 13 composite videos (one for each timing condition). The total task time was approximately 26 minutes.

Videos were displayed centered on a 20-inch (50.8-cm) computer monitor (refresh rate of 60 Hz noninterlaced). Video frames were 24-bit color images, 640 × 480 pixels in resolution. Video frame rate of presentation was 30 frames per second. No audio track accompanied the experimental stimuli.

### Apparatus and experimental setting

Eye-tracking data were collected using a dark pupil-corneal reflection video-oculography technique, with hardware and software created by ISCAN (Woburn, MA). The system was mounted on the bill of a baseball cap that participants wore throughout the session. Head movement was not restrained, but participants were told to remain as still as possible. Participants sat in a dark room, 25 inches from a 20-inch computer screen, which was mounted flush within a white wall panel. Prior to data collection, a calibration routine was performed using a 5-point calibration scheme, as in Shultz *et al*. (2011). Calibration was verified periodically throughout the experiment by presenting the 5 calibration points onscreen. Data were collected at 60 samples per second and down-sampled to the rate of stimulus presentation (30 samples per second) for analysis.

### Task & procedure

Eye-tracking data were collected while participants watched the 13 composite videos. Eleven of the 21 participants, selected at random, were instructed to count the number of *land* animals while the other 10 participants were instructed to count the number of *water* animals.

Each video consisted of three parts: an instruction screen, the composite video, and a response screen (see Fig. 1). At the beginning of each video, participants saw a 10 second instruction screen, identifying the target animal to be counted in the upcoming video. The instructions displayed both the name and a picture of the target animal. Participants then saw a composite video that alternated between scenes of animals on land and scenes of animals under water; scenes alternated at a constant rate of either 1, 5, 10, 15, 20, 25, 30, 35, 40, 45, 50, 55, or 60 s, for a total of 13 timing conditions. After each composite video, participants saw a 10 second response screen with static text that prompted them to verbally report how many target animals they counted. Participants were instructed to continue looking at the video screen while making a verbal response.

The 13 composite videos were presented in random order. Eleven of the participants saw videos in which a land scene was presented first, and the other 10 participants saw videos in which a water scene was presented first. Scene order was counterbalanced between land counters and water counters.

### Data processing

Eye tracking data were processed using in-house software that identified saccades, blinks, and off-screen fixations (fixations directed away from the stimuli presentation screen). Blinks were identified by an automated algorithm measuring occlusion of the pupil by rate of change in pupil diameter and by vertical displacement of the measured pupil center. This algorithm was previously verified through manual coding of video data in a sample of toddlers and through simultaneous video and electromyography (EMG) recording in one adult viewer^26^. In comparison with video recordings, the algorithm accurately detected 95.0% of all blinks identified by manual coding of video images. In comparison with EMG recordings, the algorithm accurately detected 96.4% of blinks recorded by EMG. Duration measurements comparing blinks detected by the algorithm and blinks detected by EMG were different by less than 10 ms (i.e., less than the sampling detection threshold of the eye-tracker). All 13 videos were included in analyses.

## Supplementary information

Supplementary Information.

## References

[1] Rose, D, H. & Gravel, J. *Universal Design for Learning Guidelines Version 2.0*. (CAST, 2011).

[2] Simons DJ, Chabris CF (1999). Gorillas in our midst: Sustained inattentional blindness for dynamic events. Perception.

[3] Council, N. R. *Educating children with autism*. (National Academies Press, 2001).

[4] Bagatell N (2012). Engaged moments: Mediated action and children with autism in the classroom setting. OTJR: Occupation, Participation and Health.

[5] Fredricks JA, Blumenfeld PC, Paris AH (2004). School engagement: Potential of the concept, state of the evidence. Review of educational research.

[6] O’Connor DH, Fukui MM, Pinsk MA, Kastner S (2002). Attention modulates responses in the human lateral geniculate nucleus. Nature neuroscience.

[7] Yi D-J, Chun MM (2005). Attentional Modulation of Learning-Related Repetition Attenuation Effects in Human Parahippocampal Cortex. The Journal of Neuroscience.

[8] Ahissar, M. & Hochstein, S. The role of attention in learning simple visual tasks (2002).

[9] Grelotti DJ (2005). fMRI activation of the fusiform gyrus and amygdala to cartoon characters but not to faces in a boy with autism. Neuropsychologia.

[10] Itti L, Koch C (2001). Computational modelling of visual attention. Nature reviews neuroscience.

[11] Yantis S, Egeth HE (1999). On the distinction between visual salience and stimulus-driven attentional capture. Journal of Experimental Psychology: Human Perception and Performance.

[12] Mazer JA, Gallant JL (2003). Goal-related activity in V4 during free viewing visual search: Evidence for a ventral stream visual salience map. Neuron.

[13] Treue S (2003). Visual attention: the where, what, how and why of saliency. Current Opinion in Neurobiology.

[14] Henderson, J. M., Brockmole, J. R., Castelhano, M. S. & Mack, M. Visual saliency does not account for eye movements during visual search in real-world scenes. *Eye movements: A window on mind and brain* 537–562 (2007).

[15] Evinger C (1995). A brain stem reflex in the blink of an eye. Physiology.

[16] Kevin O’Regan J, Deubel H, Clark JJ, Rensink RA (2000). Picture Changes During Blinks: Looking Without Seeing and Seeing Without Looking. Visual Cognition.

[17] Volkmann FC (1986). Human visual suppression. Vision research.

[18] Hoppe, D., Helfmann, S. & Rothkopf, C. A. Humans quickly learn to blink strategically in response to environmental task demands. 1–6, 10.1073/pnas.1714220115 (2018). 10.1073/pnas.1714220115 PMC5834680 29444860

[19] Nakano T, Yamamoto Y, Kitajo K, Takahashi T, Kitazawa S (2009). Synchronization of spontaneous eyeblinks while viewing video stories. Proceedings of the Royal Society B: Biological Sciences.

[20] Nakano T, Kitazawa S (2010). Eyeblink entrainment at breakpoints of speech. Experimental brain research. Experimentelle Hirnforschung. Expérimentation cérébrale.

[21] Oh J, Jeong S-Y, Jeong J (2012). The timing and temporal patterns of eye blinking are dynamically modulated by attention. Human movement science.

[22] Fukuda K, Stern JA, Brown TB, Russo MB (2005). Cognition, blinks, eye-movements, and pupillary movements during performance of a running memory task. Aviation Space and Environmental Medicine.

[23] Siegle, G. J., Ichikawa, N. & Steinhauer, S. Blink before and after you think: Blinks occur prior to and following cognitive load indexed by pupillary responses. **45**, 679–687 (2008). 10.1111/j.1469-8986.2008.00681.x 18665867

[24] Pivik, R. T. & Dykman, R. A. Endogenous eye blinks in preadolescents: relationship to information processing and performance. **66**, 191–219 (2004). 10.1016/j.biopsycho.2003.10.005 15099694

[25] Baumstimler Y, Parrot J (1971). Stimulus generalization and spontaneous blinking in man involved in a voluntary activity. Journal of Experimental Psychology.

[26] Shultz S, Klin A, Jones W (2011). Inhibition of eye blinking reveals subjective perceptions of stimulus salience. Proceedings of the National Academy of Sciences.

[27] Kanner L (1943). Autistic disturbances of affective contact. Nervous child.

[28] Chawarska K, Macari S, Shic F (2013). Decreased spontaneous attention to social scenes in 6-month-old infants later diagnosed with autism spectrum disorders. Biological psychiatry.

[29] Jones W, Carr K, Klin A (2008). Absence of preferential looking to the eyes of approaching adults predicts level of social disability in 2-year-old toddlers with autism spectrum disorder. Archives of General Psychiatry.

[30] Rice K, Moriuchi JM, Jones W, Klin A (2012). Parsing heterogeneity in autism spectrum disorders: visual scanning of dynamic social scenes in school-aged children. Journal of the American Academy of Child & Adolescent Psychiatry.

[31] Klin A, Lin DJ, Gorrindo P, Ramsay G, Jones W (2009). Two-year-olds with autism orient to non-social contingencies rather than biological motion. Nature.

[32] Dawson G, Meltzoff A, Osterling J, Rinaldi J, Brown E (1998). Children with Autism Fail to Orient to Naturally Occurring Social Stimuli. Journal of Autism and Developmental Disorders.

[33] Pierce K, Conant D, Hazin R, Stoner R, Desmond J (2011). Preference for geometric patterns early in life as a risk factor for autism. Archives of General Psychiatry.

[34] Groen Y, Borger N, Koerts J, Thome J, Tucha J (2017). Blink rate and blink timing in children with ADHD and the influence of stimulant medication. J Neural Transm.

[35] Oh J, Han M, Peterson BS, Jeong J (2012). Spontaneous eyeblinks are correlated with responses during the Stroop task. PloS one.

[36] Shin YS (2015). Correlation between inter-blink interval and episodic encoding during movie watching. PloS one.

[37] Yorzinski, J. L. Eye blinking in an avian species is associated with gaze shifts. *Scientific Reports* **6** (2016). 10.1038/srep32471 PMC5004160 27572457

[38] Ballesta, S., Mosher, C. P., Szep, J., Fischl, K. D. & Gothard, K. M. Social determinants of eyeblinks in adult male macaques. *Nature Publishing Group* 1–8, 10.1038/srep38686 (2016). 10.1038/srep38686 PMC5138631 27922101

[39] Fredericks, J. A., Blumenfeld, P. C. & Paris, A. H. School engagement: potential of the concept state of the evidence, **74**(1), 59–109 (2004).

[40] VanderWerf F, Brassinga P, Reits D, Aramideh M, Ongerboer de Visser B (2003). Eyelid Movements: Behavioral Studies of Blinking in Humans Under Different Stimulus Conditions. Journal of Neurophysiology.

[41] Dalmaso, M., Galfano, G., Coricelli, C. & Castelli, L. Temporal dynamics underlying the modulation of social status on social attention. *PLoS One* **9** (2014). 10.1371/journal.pone.0093139 PMC3965511 24667700

[42] Dzhelyova M, Jacques C, Rossion B (2016). At a Single Glance: Fast Periodic Visual Stimulation Uncovers the Spatio-Temporal Dynamics of Brief Facial Expression Changes in the Human Brain. Cerebral Cortex.

[43] Doughty MJ (2002). Further assessment of gender-and blink pattern-related differences in the spontaneous eyeblink activity in primary gaze in young adult humans. Optometry & Vision Science.

[44] Briggs ST (1999). Spontaneous blink rate of a normal population sample. International Contact Lens Clinic.

[45] Sforza C, Rango M, Galante D, Bresolin N, Ferrario VF (2008). Spontaneous blinking in healthy persons: an optoelectronic study of eyelid motion. Ophthalmic and Physiological Optics.

[46] Bentivoglio AR (1997). Analysis of blink rate patterns in normal subjects. Movement Disorders.

[47] Holm, S. A simple sequentially rejective multiple test procedure. *Scandinavian journal of statistics* 65–70 (1979).

[48] Hanley JA, McNeil BJ (1982). The meaning and use of the area under a receiver operating characteristic (ROC) curve. Radiology.

[49] Agresti, A., Coull, B. A., Statistician, T. A. & May, N. Agresti_Coull_1998-1. **52**, 119–126 (2007).

[50] Cortes C, Vapnik V (1995). Support-vector networks. Machine learning.

[51] Picard RR, Cook RD (1984). Cross-validation of regression models. Journal of the American Statistical Association.

[52] Ben-Hur, A. & Weston, J. A user’s guide to support vector machines. in *Data mining techniques for the life sciences* 223–239 (Springer, 2010). 10.1007/978-1-60327-241-4_13 20221922

[53] Hsu, C.-W., Chang, C.-C. & Lin, C.-J. A practical guide to support vector classification (2003).

[54] Good, P. *Permutation, Parametric and Bootstrap Tests of Hypotheses*. (Springer Verlag, 2005).

[55] Doughty MJ (2001). Consideration of three types of spontaneous eyeblink activity in normal humans: during reading and video display terminal use, in primary gaze, and while in conversation. Optometry and Vision Science.

[56] Parzen E (1962). On estimation of a probability density function and mode. The annals of mathematical statistics.

[57] Iovannone R, Dunlap G, Huber H, Kincaid D (2003). Effective educational practices for students with autism spectrum disorders. Focus on autism and other developmental disabilities.

[58] Ruble LA, Robson DM (2007). Individual and environmental determinants of engagement in autism. Journal of autism and developmental disorders.

[59] Sparapani N, Morgan L, Reinhardt VP, Schatschneider C, Wetherby AM (2016). Evaluation of Classroom Active Engagement in Elementary Students with Autism Spectrum Disorder. Journal of Autism and Developmental Disorders.

[60] Sasson NJ, Turner‐Brown LM, Holtzclaw TN, Lam KSL, Bodfish JW (2008). Children with autism demonstrate circumscribed attention during passive viewing of complex social and nonsocial picture arrays. Autism Research.

[61] Green MF (2008). Social cognition in schizophrenia: an NIMH workshop on definitions, assessment, and research opportunities. Schizophrenia bulletin.

[62] García-Blanco A, Salmerón L, Perea M (2015). Attentional capture by emotional scenes across episodes in bipolar disorder: Evidence from a free-viewing task. Biological psychology.

[63] De Raedt R, Koster EHW (2010). Understanding vulnerability for depression from a cognitive neuroscience perspective: A reappraisal of attentional factors and a new conceptual framework. Cognitive, Affective, & Behavioral Neuroscience.

