# Supplementary material for: Blink Rate Patterns Provide a Reliable Measure of Individual Engagement with Scene Content
Source: Sci Rep. 2020 May 19;10:8267. doi: 10.1038/s41598-020-64999-x (PMC7237680; doi:10.1038/s41598-020-64999-x)
Supplement: Supplementary file 1 — Supplementary Information. [file 41598_2020_64999_MOESM1_ESM.docx]

**Blink Rate Patterns Provide a Reliable Measure of Individual Engagement with Scene Content**

Carolyn Ranti^1,2^, Warren Jones^1,2,3^, Ami Klin^1,2,3^, & Sarah Shultz*^1,2^

^1^ Marcus Autism Center, Children’s Healthcare of Atlanta, Atlanta, Georgia 30329, USA.

^2^ Division of Autism & Related Disabilities, Department of Pediatrics, Emory University School of Medicine, Atlanta, Georgia 30022, USA.

*^3^  Center for Translational Social Neuroscience, Emory University, Atlanta, Georgia 30022, USA.*

Correspondence to sarah.shultz@emory.edu

**Supplementary Materials**

**Supplementary Figure 1. Relationship between individual blink rate and classification strength for scenes of varying length.** No significant relationship was observed between individual blink rate and log likelihood ratio (log(LR)) for 5 to 60 second scenes. Only the linear regression for the shortest scene duration (1s) was significant (r^2^=0.735, *p*= 6.8319E-7, Bonferroni for multiple comparisons (13 total)) (see **Figure 1** in main manuscript). Although a trend towards a significant relationship can be observed for each scene duration, our classification results show that even individuals with very low blink rates (e.g. 2 bpm) can be classified successfully on the basis of blink rate patterns.

**Supplementary Table 1**: Linear versus Deming Regression estimates and 95% confidence intervals

| **Interval Size** | **Deming Regression** | | **Linear Regression** | |
| --- | --- | --- | --- | --- |
|  | **Estimate** | **95% CI** | **Estimate** | **95% CI** |
| 1 second |  |  |  |  |
| Intercept | -0.523 | (-1.198, 0.152) | -0.520 | (-1.319, 0.279) |
| Individual BR | 0.235 | (0.118, 0.353) | 0.235 | (0.167, 0.303) |
|  |  |  |  |  |
| 5 seconds |  |  |  |  |
| Intercept | -0.026 | (-0.751, 0.698) | -0.024 | (-1.217, 1.168) |
| Individual BR | 0.138 | (0.045, 0.231) | 0.138 | (0.064, 0.212) |
|  |  |  |  |  |
| 10 seconds |  |  |  |  |
| Intercept | 1.271 | (-0.307, 2.849) | 1.275 | (-0.554, 3.105) |
| Individual BR | 0.064 | (-0.093, 0.221) | 0.064 | (-0.067, 0.194) |
|  |  |  |  |  |
| 15 seconds |  |  |  |  |
| Intercept | 0.956 | (-0.125, 2.040) | 0.958 | (-0.330, 2.247) |
| Individual BR | 0.055 | (-0.016, 0.124) | 0.054 | (-0.031, 0.139) |
|  |  |  |  |  |
| 20 seconds |  |  |  |  |
| Intercept | 0.607 | (-0.117, 1.339) | 0.615 | (-1.250, 2.480) |
| Individual BR | 0.047 | (-0.054, 0.147) | 0.046 | (-0.095, 0.187) |
|  |  |  |  |  |
| 25 seconds |  |  |  |  |
| Intercept | 0.021 | (-0.351, 0.395) | 0.022 | (-0.965, 1.010) |
| Individual BR | 0.020 | (-0.025, 0.065) | 0.020 | (-0.038, 0.078) |
|  |  |  |  |  |
| 30 seconds |  |  |  |  |
| Intercept | -1.934 | (-5.481, 1.663) | -1.741 | (-4.800, 1.318) |
| Individual BR | 0.286 | (-0.151, 0.718) | 0.270 | (0.062, 0.478) |
|  |  |  |  |  |
| 35 seconds |  |  |  |  |
| Intercept | 0.038 | (-0.620, 0.702) | 0.043 | (-2.079, 2.166) |
| Individual BR | 0.038 | (-0.056, 0.132) | 0.038 | (-0.083, 0.159) |
|  |  |  |  |  |
| 40 seconds |  |  |  |  |
| Intercept | -58.707 | (-188.131, 65.172) | -12.754 | (-30.045, 4.537) |
| Individual BR | 5.438 | (-5.609, 16.997) | 1.582 | (0.350, 2.814) |
|  |  |  |  |  |
| 45 seconds |  |  |  |  |
| Intercept | -6.966 | (-24.643, 11.534) | -2.798 | (-8.837, 3.240) |
| Individual BR | 0.667 | (-0.960, 2.234) | 0.358 | (-0.031, 0.740) |
|  |  |  |  |  |
| 50 seconds |  |  |  |  |
| Intercept | -0.602 | (-2.632, 1.435) | -0.588 | (-2.548, 1.373) |
| Individual BR | 0.119 | (-0.136, 0.373) | 0.118 | (-0.017, 0.253) |
|  |  |  |  |  |
| 55 seconds |  |  |  |  |
| Intercept | 0.207 | (-0.458, 0.878) | 0.212 | (-1.208, 1.632) |
| Individual BR | 0.051 | (-0.066, 0.168) | 0.051 | (-0.060, 0.161) |
|  |  |  |  |  |
| 60 seconds |  |  |  |  |
| Intercept | 0.690 | (-0.268, 1.645) | 0.689 | (-0.473, 1.851) |
| Individual BR | 0.002 | (-0.055, 0.059) | 0.002 | (-0.090, 0.095) |

^1^Deming Regression confidence interval derived via Jackknife
